# Long-Term Outcome of Single-Session, Ultrasound-Guided, Radiofrequency Ablation for Symptomatic Small, Lower Limb, Venous Malformations

**DOI:** 10.5334/jbsr.2801

**Published:** 2022-07-13

**Authors:** Laurence Verhaeghe, Veerle Labarque, Jan Vranckx, Inge Fourneau, Steven Pans, Geert Maleux

**Affiliations:** 1 Department of Radiology, University Hospitals KU Leuven, Leuven, BE; 2 Department of Pediatrics, University Hospitals KU Leuven, Leuven, BE; 3 Department of Plastic and Reconstructive Surgery, University Hospitals KU Leuven, Leuven, BE; 4 Department of Vascular Surgery, University Hospitals KU Leuven, Leuven, BE

**Keywords:** vascular malformation, lower limb, radiofrequency ablation, percutaneous, outcome

## Abstract

**Objectives:**

To analyze the long-term clinical outcome of percutaneous, ultrasound-guided radiofrequency ablation (RFA) of peripheral low-flow vascular malformations (VM).

**Materials & Methods:**

Adolescent patients presenting with symptomatic VMs and referred for percutaneous management using RFA between January 2010 and January 2015 were identified by a search in the institutional interventional radiology database. Clinical and radiological follow-up up to April 2021 was based on retrospective analysis of patients’ electronic medical records and imaging files. This retrospective study was approved by the institutional ethics committee.

**Results:**

Four female patients (median age 16 years) presented with lower extremity pain, swelling and functional disability related to VM as confirmed by magnetic resonance imaging. Two patients underwent percutaneous sclerotherapy previously. Clinical follow-up (mean of 8,5 years) showed complete and sustained resolution of the symptoms in all patients; in one patient a persistent decrease in muscular strength of the treated limb was noted.

**Conclusion:**

Percutaneous, ultrasound-guided RFA is relatively safe and efficient with durable symptom relief in the management of small, lower limb VMs in adolescent patients.

## Introduction

Venous malformations (VMs) are congenital, developmental anomalies which might occur anywhere in the body, but typically seen in the head and neck region (40%), extremities (40%) and trunk (20%) [1]. Symptoms may vary, depending on extent and location of the VM, and may include swelling, pain, bleeding, muscle motion restriction and contracture and thrombophlebitis [2].

Management of VMs traditionally includes compression therapy, percutaneous sclerotherapy and debulking surgery [3,4,5]. Percutaneous sclerotherapy, using various types of liquid sclerosants, is not free of complications, including tissue ischemia and infarction, nerve injury, muscle contracture, and skin ulceration in up to 13% of cases [6]. In addition, patient satisfaction is around 50% at five years of follow-up [7]. Recently, a few case reports and small case series reported the feasibility on safety of percutaneous, image-guided ablation of small intramuscular and/or subcutaneous VM using both radiofrequency ablation (RFA) [8,9,10,11] as well as cryoablation [12]. In this report we retrospectively evaluated the safety and long-term clinical outcome of small, symptomatic VM in adolescents treated with percutaneous, single-session radiofrequency ablation.

## Materials and Methods

### Study design & study population

This is a retrospective study, including consecutive adolescent patients presenting with a symptomatic extra-troncular low flow venous malformation, located in the subcutaneous and/or intramuscular region of the extremities and treated with RFA between April 2010 & January 2015. Patients were seen at the institutional multidisciplinary clinic for vascular malformations including different specialties: interventional radiology, vascular medicine, pediatrics, plastic & reconstructive surgery, vascular surgery, and maxillofacial surgery. Data of patients’ history and characteristics, indication for referral to percutaneous ablation therapy and follow-up data were gathered from the electronic medical records, and the hospital Picture Archiving and Communicating System (PACS, Agfa Gevaert, Mortsel, Belgium).

This retrospective study was approved by the institutional ethics committee and all patients gave informed consent before percutaneous ablation procedure.

### Radiological Assessment at Baseline and During Follow-Up

Soft tissue ultrasound was performed using a 4–15 MHz array linear probe to localize and differentiate the VM. In addition, magnetic resonance imaging of the VM was performed, including T2 fat saturated images (STIR/SPAIR/SPIR) as well as T1-weigthed axial and coronal images without and with 15 ml of Gadolinium-contrast medium (gadoteric acid-gadoterate meglumine, Dotarem, Guerbet, Villepinte, France).

### Percutaneous Radiofrequency Ablation Procedure

Procedures were performed under general anesthesia. After draping the affected limb, the VM was punctured percutaneously under ultrasound guidance using various types of RFA needles (Cool-Tip RF Electrode Kit, Valleylab, Tyco, Boulder, CO, USA or StarBurst, Angiodynamics, Queensbury, NY, USA). Conventionally, the needle tips were placed in the central portion of the lesion and depending on the extent of the lesion one or more needles were inserted in accordance with the manufacturer’s instructions for use. Ablation time varied between four and six minutes with needle tip temperatures reached in between 85° and 100°.

### Patients’ Follow-Up

Patients were followed-up clinically at 1, 6 and 12 months or in case of adverse events. Late clinical follow-up was performed by telephone calls.

In case of residual or persistent complaints, additional follow-up magnetic resonance imaging was performed using T2 fat saturated imaging and T1 imaging without intravenous injection of Gadolinium contrast medium.

## Results

Patients were female and in between 11 and 20 years of age (median 16 years); the majority of patients presented with an intramuscular symptomatic VM with a median diameter of 2.9 cm at the lower limb (Figure 1a); two out of four patients previously underwent unsuccessful percutaneous sclerotherapy as summarized in Table 1.

**Figure 1a:**
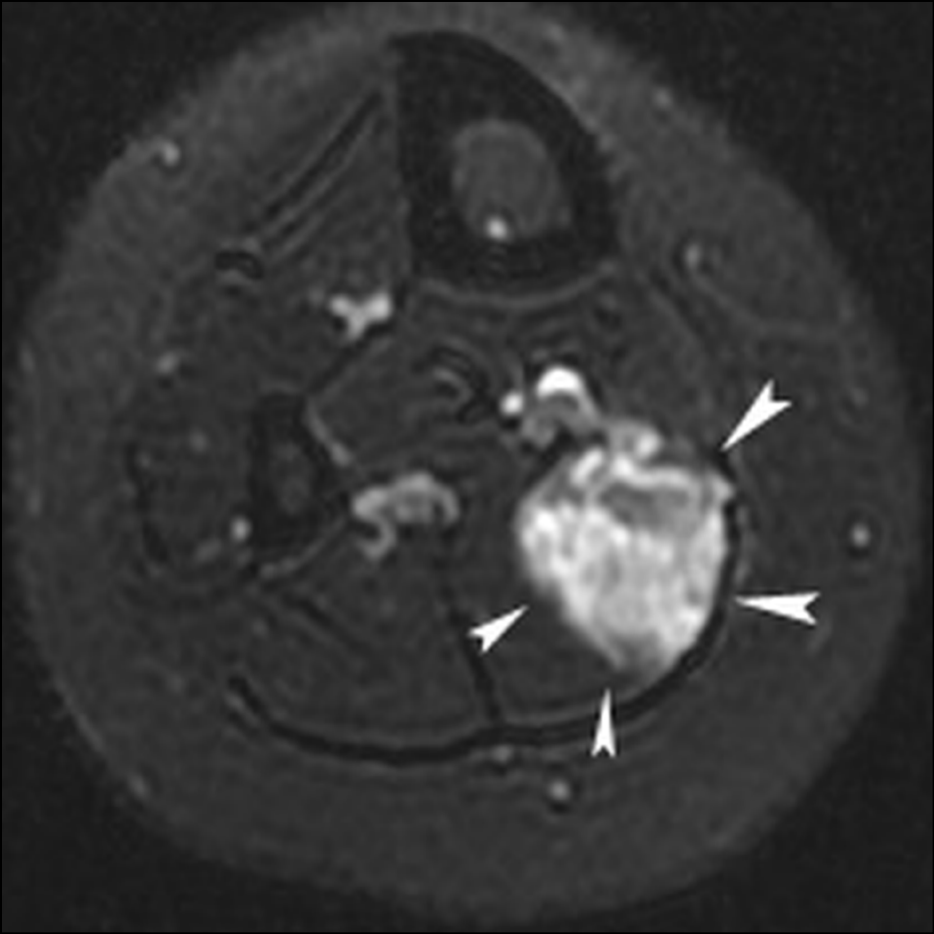
Twenty-year-old female with chronic calf pain. T2-weigthed axial magnetic resonance image of the calf demonstrating an hyperintense mass lesion with a maximal diameter of 3.6 cm (white arrowheads) in the right soleus muscle, suggestive for low-flow vascular malformation.

**Table 1:** Patients demographics, clinical and radiological presentation of the VM.


D/C/R PARAMETER	PATIENT 1	PATIENT 2	PATIENT 3	PATIENT 4

Age	11 y	14 y	20 y	19 y

Sex	female	female	female	female

Location of VM: Lower extremity				

upper leg	–	–	–	+

lower leg	–	+	+	–

Foot	+	–	–	–

Intramuscular	–	+	+	+

	(lateral gastrocnemius) (soleus) (vastus intermedius)			

Subcutaneous tissue	+	–	–	–

Maximum diameter of the VM	2 cm	1, 8 cm	3, 6 cm	4, 3 cm

Patients’ symptoms:				

Local pain	+	+	+	+

Local swelling	+	+	+	+

Functional disability	+	+	-	+

Duration of symptoms since onset	NM	4 y	3 y	3 y

Previous treatment				

sclerotherapy	–	+	+	–

. ethanol 96%	–	+	–	–

. sotradecol 3%	–	+	+ (2x)	–

Interval between last sclerotherapy and RFA				

	–	8 months	8 months	–


VM = venous malformations. RFA = radiofrequency ablation. NM = not mentioned in electronic medical record.

Clinical follow-up at 1 and 6 months revealed complete resolution of the swelling symptoms in all patients; resolution of pain was observed in three out of four patients and less invalidating pain in the remaining patient as summarized in Table 2. One patient presented with new onset of partial loss of muscle strength over the first six months after the index RFA. Radiological re-evaluation with magnetic resonance imaging, performed three months after RFA, demonstrated in this patient complete disappearance of the ablated VM (Figure 1b).

**Table 2:** Patients’ clinical short- and long-term follow-up.


PARAMETER	PATIENT 1	PATIENT 2	PATIENT 3	PATIENT 4

Short & midterm FU (1 year)				

Local pain	–	–	–	–

Local swelling	–	–	–	–

Functional disability	–	–	+	–

Procedure-related complication	–	–	+	–

Long-term follow-up (until 1/4/2021)				

FU interval (years)	10 y	11 y	7 y	6 y

Local pain	–	–	–	–

Local swelling	–	–	–	–

Functional disability	–	–	–/+	–

Procedure-related complication	–	–	+	–


**Figure 1b:**
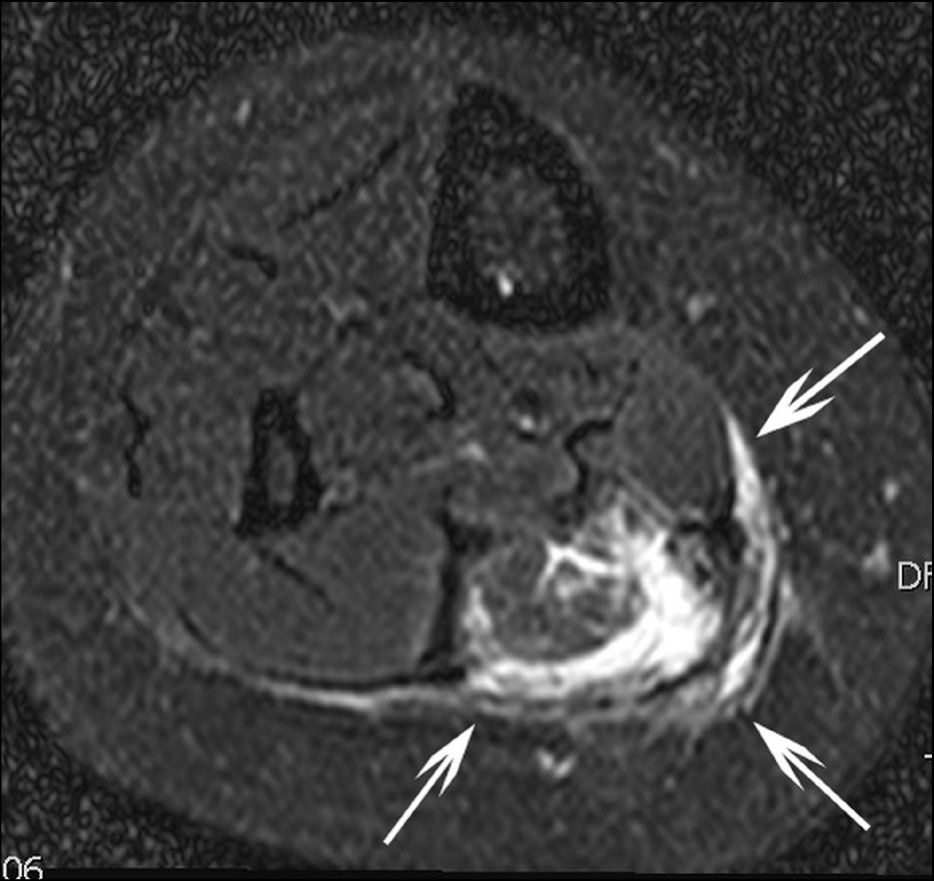
T2-weigthed axial magnetic resonance image in the same patient three months after percutaneous radiofrequency ablation which was performed after two failed percutaneous sclerotherapies with sotradecol. Hyperintense rim (white arrows) around the ablated area suggestive for perileasional oedema.

Mid-term (6–12 months) and long-term (median 8.5 years) clinical follow-up showed complete resolution of pain, swelling and functional disability in all patients; the residual, partial, post-ablation loss of muscle strength in one patient decreased during follow-up owing to several months of intensive physiotherapy.

## Discussion

This report demonstrates the feasibility of percutaneous RFA for small, lower limb intramuscular and subcutaneous VMs, which is in line with the findings of van der Linden et al. demonstrating high short-term clinical success after RFA for small VMs [8]. In addition, RFA seems to be a valuable treatment option for VMs, not only after failed sclerotherapy [8], but also as a first treatment option. This might be an important advantage compared to percutaneous sclerotherapy, which typically needs several sessions before resulting in meaningful clinical success: Bianchini et al. performed on average two to three sessions of sclerotherapy using polidocanol or ethanol as sclerosant agent [13]; van der Linden et al. found a 42% of clinical failures at three months of follow-up using the same sclerosant agents for sclerotherapy [7].

Treatment of small VMs with percutaneous RFA is not free of adverse events. We encountered in one patient local nerve injury resulting in partial loss of muscle strength, which partially recovered after several months and several sessions of physiotherapy. Systemic complications, including systemic ethanol contamination [14] or cardiovascular collapse due to right heart failure as sometimes seen after sclerotherapy [15], nearly seems impossible after RFA.

Last, symptom relief after RFA seems to be much more durable compared to percutaneous sclerotherapy. In the presented report, based on four patients, we did not encounter any recurrent symptoms after more than five years of follow-up, whereas van der Linden et al. found only 58% of partial or complete relief of VM complaints after a follow-up of five years [7].

## Limitations

Limitations of the report mainly include the retrospective nature of the study design and the limited number of included patients (n = 4). In addition, only one interventionalist performed the procedures with two types of radio frequency devices. Multicenter, prospective study design with various types of radiofrequency devices are required to confirm the presented, preliminary data.

## Conclusion

In conclusion, this short report, based on follow-up data of four selected cases of small, lower limb VMs, seems to suggest the durable clinical effect of percutaneous RFA for both de novo VMs and VMs after failed sclerotherapy and might suggest initiation of larger studies on RFA as the first treatment option for small (diameter less than 4 cm) intramuscular and/or subcutaneous VMs and being a real alternative to percutaneous sclerotherapy.

## Main points

- Percutaneous, ultrasound-guided radiofrequency ablation is relatively safe and effective for the treatment of small, low-flow venous malformations in the lower limb.
- Long-term clinical outcome data shows this treatment is durable.
- Peripheral nerve damage during the intervention might occur with partial recovery after several months of physiotherapy.
